# A young female presenting with unilateral sacroiliitis following dengue virus infection: a case report

**DOI:** 10.1186/s13256-017-1483-0

**Published:** 2017-11-01

**Authors:** W. D. Jayamali, H. M. M. T. B. Herath, Aruna Kulatunga

**Affiliations:** National Hospital of Sri Lanka, Colombo, Sri Lanka

**Keywords:** Dengue, Sacroiliitis, Post-viral arthritis

## Abstract

**Background:**

Dengue is a common arthropod-borne viral infection in Sri Lanka which is spread by the mosquitos of the genus Aedes. The clinical features of dengue include high-grade fever associated with arthralgia and myalgia. However, dengue virus is not considered an arthritogenic virus. We report a case of a previously healthy young female who presented with imaging-confirmed right-sided sacroiliitis 10 days after developing dengue fever. This is the first reported case that shows a possible link between dengue infection and development of arthritis.

**Case presentation:**

A 14-year-old Sri Lankan female presented to our medical unit with right buttock and hip pain of 3 weeks’ duration. She had serologically confirmed dengue infection 10 days prior to the onset of buttock pain. A clinical examination revealed features of right sacroiliitis. An X-ray of her sacroiliac joint showed joint space widening and reactive bone changes. Magnetic resonance imaging of her pelvis and sacroiliac joint confirmed the diagnosis of acute sacroiliitis. She had an erythrocyte sedimentation rate of 110 mm first hour with a normal C-reactive protein. Her human leukocyte antigen-B27, rheumatoid factor, antinuclear antibody, chikungunya antibody, hepatitis serology, Brucella serology, and tuberculin skin test were negative. She was treated with nonsteroidal anti-inflammatory drugs and showed gradual improvement.

**Conclusions:**

After excluding possible causes for sacroiliitis, we postulated that sacroiliitis in the index case could have been caused or triggered by dengue virus infection. However there is a possibility that the sacroiliitis merely coincided with the dengue virus infection. This case illustrates the possibility that dengue virus could have a link with the development of arthritis in the same manner as other arthritogenic viruses; possible mechanisms for this include direct invasion of the synovium and the joint tissue by the virus, immune complex formation and deposition in the joint tissue, and immune dysregulation. Further studies are needed in this field to gain more knowledge, as dengue infection is highly prevalent in Sri Lanka.

## Background

Dengue is a common arthropod-borne viral infection in Sri Lanka which is spread by the mosquitos of the genus Aedes: Aedes aegypti and Aedes albopictus. It has a high global prevalence with 128 countries being affected and a 3.9 billion people at risk [1]. In spite of the implementation of national dengue control methods including environmental management and legislation, and chemical and biologic vector control methods, in 2016, 55,150 cases of dengue were reported from Sri Lanka indicating the high burden of the disease [2, 3].

The clinical manifestations of dengue infection have a wide spectrum although many who are infected with the virus remain asymptomatic. The clinical syndrome can vary from mild undifferentiated fever to life-threatening dengue hemorrhagic fever and dengue shock syndrome. The virus can affect the heart, liver, and nervous system giving rise to clinical sequelae of myocarditis, hepatitis, dengue encephalopathy, and various other reported neurological complications such as Guillain–Barré syndrome, acute disseminated encephalomyelitis, and brachial neuritis [4, 5]. It can also give rise to oral manifestations such as erythema and crusting of the lips, vesicles and hemorrhagic bulla on the tongue, and buccal mucosa [6].

The fever that occurs in dengue virus infection is associated with severe arthralgia and myalgia. However, dengue virus is not considered an arthritogenic virus. Arthritogenic viruses include rubella virus, hepatitis B and C, parvovirus, and alphaviruses such as chikungunya virus [7]. Dengue virus is a flavivirus and so far not reported to cause arthritis.

We report a case of a previously healthy young girl who presented with imaging-confirmed right-sided sacroiliitis 10 days after developing dengue fever. To the best of our knowledge this is the first reported case that shows a possible link between dengue infection and development of arthritis.

## Case presentation

A 14-year-old Sri Lankan girl presented to our medical unit with right-sided buttock pain and hip pain of 3 weeks’ duration. She had been in good health before she developed fever, arthralgia, myalgia, and headache approximately 5 weeks earlier. A nonstructural protein 1 (NS1) antigen test for dengue fever had been positive and hematological and biochemical investigations were compatible with dengue fever. She was treated at a local hospital where she had an uneventful recovery and was discharged after 6 days. Ten days after the onset of fever (4 days after the discharge) she developed right-sided buttock and hip pain with difficulty in walking. She was readmitted to the local hospital and was managed as post-viral arthritis. She was treated with nonsteroidal anti-inflammatory drugs (NSAIDs) and steroids. Her symptoms improved and she was discharged home. However, her symptoms recurred after a few days and had persisted with worsening pain.

She did not have other small or large joint pain or swelling and there was no history suggestive of inflammation of enthesitis. She did not have fever during this episode. She did not have red eyes, dysuria, or skin eruptions. There was no preceding history of a diarrheal illness, sore throat, or any illness other than dengue fever. There was no past history of joint pains, recurrent oral ulceration, or photosensitive rashes. There was no history of altered bowel habits or bloody diarrhea suggestive of inflammatory bowel disease. There was no family history of arthritis or autoimmune diseases. There was no past history or contact history of tuberculosis.

On examination she was mildly pale. There was no uveitis, oral ulceration, or skin lesions. Her cardiovascular system, respiratory system, and abdominal examination were normal. There was tenderness over her sacroiliac joint and sacroiliac stretch maneuvers were positive suggestive of right-sided sacroiliitis. Her left-side sacroiliac examination was normal. There was no tenderness over her spine and the straight leg raising sign was negative. A neurological examination of her lower limbs was normal. The rest of the joint examination was normal as well.

**Fig. 1:**
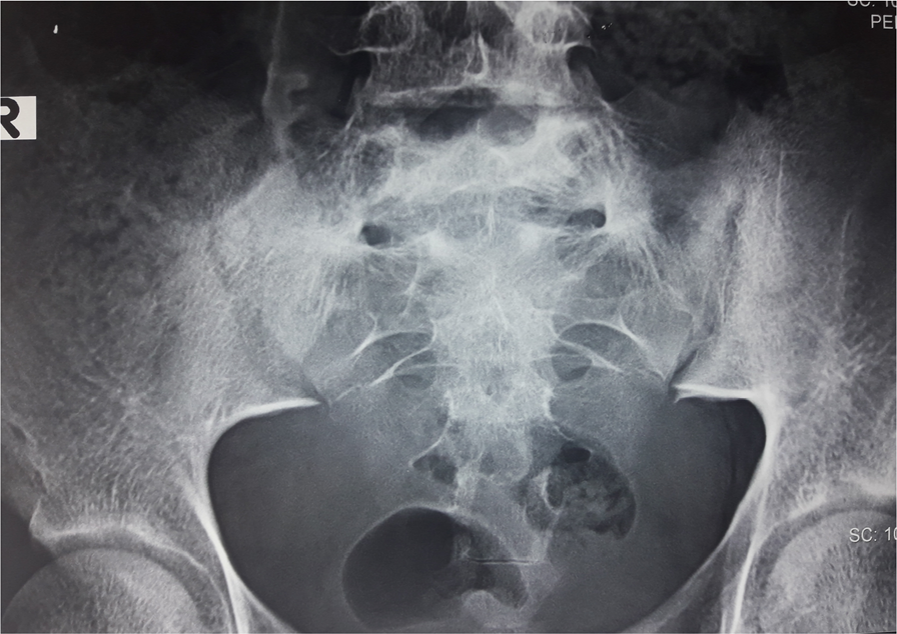
X-ray of the sacroiliac joint showing features of right-sided sacroiliitis with joint space widening and reactive bony changes surrounding the joint

**Fig. 2:**
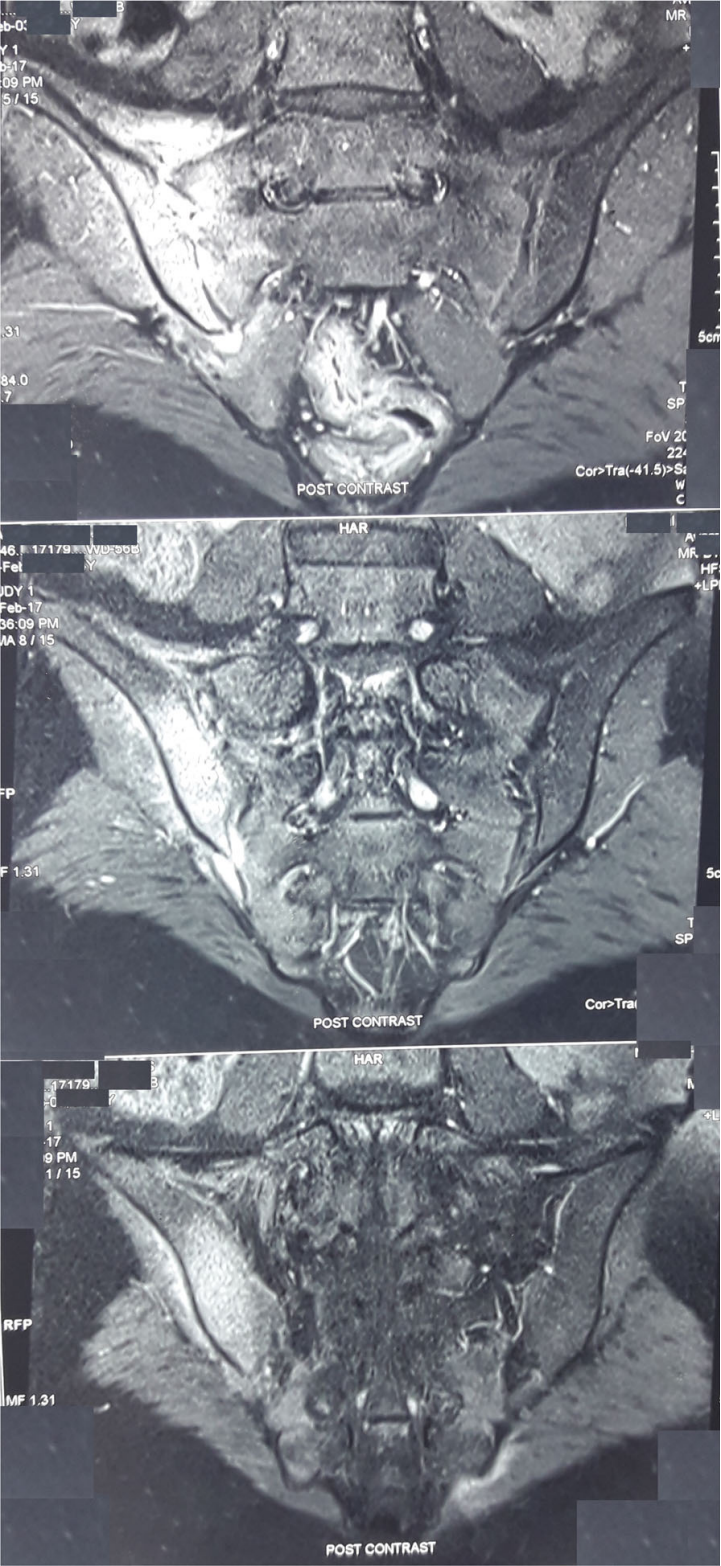
Magnetic resonance imaging of the sacroiliac joint showing high T2 signal intensity around right sacroiliac joint with surrounding marrow edema and reactive bony changes suggestive of acute right-sided sacroiliitis

An X-ray of her sacroiliac joint revealed features of right-sided sacroiliitis with joint space widening and reactive bony changes surrounding the joint (Fig. 1). Magnetic resonance imaging (MRI) of her sacroiliac joint revealed evidence of right sacroiliac joint inflammation with surrounding marrow edema and reactive bony changes suggestive of acute right-sided sacroiliitis (Fig. 2).

Her complete blood count showed neutrophil leukocytosis and a microcytic hypochromic anemia (Table 1). Blood picture showed microcytic hypochromic red blood cells and a neutrophil leukocytosis without toxic neutrophil changes. Liver functions, renal functions, uric acid level, and thyroid functions were normal (Table 1). Her erythrocyte sedimentation rate (ESR) was 110 mm first hour and C-reactive protein (CRP) was less than 6 mg/dl. Blood cultures and urine cultures were negative. Tuberculin skin test and Brucella serology and cultures were negative. Dengue immunoglobulin M (IgM) done during the current admission was positive. Chikungunya antibody was negative. Human leukocyte antigen (HLA)-B27, rheumatoid factor, antinuclear antibody (ANA), hepatitis A serology, hepatitis B serology, hepatitis C serology, human immunodeficiency virus (HIV) serology, Epstein–Barr virus (EBV) serology, cytomegalovirus (CMV) serology, as well as HLA-B51 were negative.

**Table 1:** Basic hematological and biochemical investigations

Investigation and value	Reference range	Investigation and value	Reference range
WBC 16.03 × 10^3^/μL	4–10	Hemoglobin 9.1 g/dL	11–16
Lymphocyte 4.58 × 10^3^/μL	0.8–4.0	Mean corpuscular volume 75.1 fL	80–100
Neutrophils 10.10 × 10^3^/μL	2–7	Mean corpuscular hemoglobin 23.1 pg	27–34
Platelets 402 × 10^3^/μL	150–450		
Serum creatinine 72 μmol/l	60–120	Serum potassium 4.5 mmol/L	3.5–5.1
Sodium 137 mmol/L	135–148		
AST 17 U/L	<35	ALT 25 U/L	<35
Alkaline phosphatase 114 U/L	50–162	Total bilirubin 7.9 μmol/L	
Direct bilirubin 1.6 μmol/L	<3.4	Total protein 68 g/L	
Albumin 33 g/L	36–50	Globulin 35 g/L	
Uric acid 3.4 mg/dL	2.4–6.1		
Free T4 1.37	0.6–4.5	TSH 1.92	0.8–2
Serum iron 35.8	37–145	TIBC 427	274–497
Transferring saturation 9.3%	20–50	Serum ferritin 25 ng/mL	28–365

ALT alanine aminotransferase, AST aspartate aminotransferase, T4 thyroxin, TIBC total iron-binding capacity, TSH thyroid-stimulating hormone, WBC white blood cell.

Iron studies revealed iron deficiency status (Table 1) and she was started on iron supplementation with orally administered ferrous sulfate (FeSO4) 200 mg three times a day and vitamin C 1 tablet three times a day which was continued for a duration of 6 months. She was seen by the rheumatology team and the diagnosis of right sacroiliitis was confirmed. She was treated with orally administered diclofenac sodium 50 mg every 8 hours and acetaminophen for the pain. Her symptoms gradually improved with NSAIDs and her ESR reduced to 70 from 110 after 1.5 weeks of treatment. Physiotherapy was arranged at the local hospital and she is to be followed up at regular intervals in our unit as well as at the rheumatology clinic.

## Discussion

Dengue fever usually manifests with significant musculoskeletal symptoms including arthralgia and myalgia. However, dengue virus as a cause of arthritis is not reported so far. Our patient was a previously healthy girl who developed serologically proven uncomplicated dengue fever and later on developed imaging-proven right sacroiliitis 10 days after the onset of dengue. Whether the dengue was the cause or the triggering factor for the sacroiliitis or whether it was an entirely different pathology that merely coincided with the dengue is a dilemma. However, the fact that there was no preceding history of spondyloarthropathy and most of the known causes of sacroiliitis were excluded raises the possibility that sacroiliitis may be linked with dengue.

Sacroiliitis can occur in isolation due to infectious causes or as part of an inflammatory arthropathy, mainly seronegative spondyloarthropathy. The most common bacterial infections that have a predilection for sacroiliac joints are brucellosis and tuberculosis, which usually cause unilateral disease [8, 9]. Non-brucellosis and non-tuberculous infective sacroiliitis is rare, yet staphylococci and Pseudomonas aeruginosa infections were among the most frequently identified [10]. In our patient, serology for Brucella and tuberculin skin test were negative and blood cultures did not isolate any organism. She did not have fever or any other constitutional symptoms, which makes an infective etiology unlikely. Sacroiliitis is an early feature of spondyloarthropathies and this group consists of ankylosing spondylitis, psoriatic arthritis, reactive arthritis, inflammatory bowel disease-related arthritis, and undifferentiated spondyloarthropathies [11]. These conditions usually cause a bilateral symmetrical or asymmetrical arthritis. Our patient did not have a history of joint diseases, enthesitis, uveitis, conjunctivitis, urethritis, or altered bowel habits pertaining to any of the clinical entities mentioned above. Her HLA-B27 was negative as well. Other inflammatory arthritis that can give rise to sacroiliitis include Behçet’s disease, gout, and, rarely, rheumatoid arthritis [12]. However, she did not have recurrent oral ulceration or any other features that fulfil the criteria for Behçet’s disease and HLA-B51 was negative. Her serum uric acid was normal and rheumatoid factor was negative as well.

Several viruses are also implied as the causes of arthritides. Viruses can cause infection or act as cofactors in the development of rheumatic diseases. The viruses that usually cause arthritis include rubella, mumps, parvovirus B19, hepatitis B and C, EBV, CMV, and the group of arthritogenic alphaviruses such as chikungunya, Ross River virus (RRV), Barmah Forest virus, Sindbis virus, o'nyong-nyong virus, and Mayaro virus [7, 13]. However, most of these cause symmetrical polyarthritis or asymmetrical oligoarthritis. Chikungunya virus was linked to sacroiliitis in one reported case. Nisar and Packianatha described a case of a 51-year-old woman who presented with bilateral sacroiliitis 1 year after contracting chikungunya [14]. She had a very strongly positive chikungunya IgG titer and the rest of the serological investigations were negative for other possible causes of spondyloarthropathy. Therefore they postulated a possible link between chikungunya virus and the development of spondyloarthropathy. In our patient chikungunya antibody was negative as well as hepatitis, EBV, and CMV serology.

There are several hypothesized mechanisms of the pathogenesis of arthritis following viral infection. These postulated mechanisms are:

1. Direct invasion of the synovium and the joint tissue due to tropism of these viruses for the synovial tissue, for example rubella virus and vaccine, parvovirus.
2. By means of immune complex formation and deposition in the joint tissue as the viral particles act as antigenic components, for example alphaviruses, hepatitis B and C, and parvovirus.
3. Latent viruses, immune dysregulation, and virus-induced autoimmunity: where the viruses remain in the host cells expressing viral antigens on the cellular surface inducing a chronic inflammatory status or viruses directly invade the components of the immune system and cause immune dysregulation resulting in autoimmune diseases (for example HIV, human T-lymphotropic virus 1, or hepatitis C virus) [15–17]. Molecular mimicry may cause abnormal self-reactivity by altering immune tolerance [18].
4. Chen and colleagues showed that primary human osteoblasts (HObs) could be productively infected by RRV and the RRV-infected HObs produce high levels of inflammatory cytokine including interleukin-6 (IL-6) resulting in increased receptor activator of nuclear factor kappa-B ligand (RANKL) but decreased osteoprotegerin (OPG) [19]. This elevation in RANKL/OPG ratio favors osteoclast leading to an increase in bone reabsorption and bone pathologies. They also reported that inhibition of IL-6 alleviates inflammation and targeting IL-6 early by using anti-IL-6R antibodies, such as tocilizumab, will likely be therapeutically beneficial [19].

Therefore, there is a possibility that the dengue virus too can affect the joint tissues by the means of the above mechanisms, particularly the first two, and it is an area that needs to be explored. These viral arthritides are usually mild and self-limiting, lasting no longer than a few weeks. There is no specific treatment and simple symptomatic measures with analgesics are sufficient [20].

## Conclusions

Even though dengue infection causes significant musculoskeletal symptoms it was not reported to cause arthritis. Here we presented a case of a previously healthy young girl who presented with unilateral sacroiliitis following dengue virus infection. After excluding other possible causes for sacroiliitis, we postulated that sacroiliitis could have been caused or triggered by dengue fever in this patient but there is a possibility that the sacroiliitis merely coincided with the dengue fever. This case illustrates the possibility that dengue virus could have a link with development of arthritis in the same manner as other arthritogenic viruses; possible mechanisms for this include direct invasion of the synovium and the joint tissue by the virus, immune complex formation and deposition in the joint tissue, and immune dysregulation. Further studies are needed in this field to gain more knowledge, as dengue infection is highly prevalent in Sri Lanka.

## Abbreviations

ANA: Antinuclear antibody
CMV: Cytomegalovirus
CRP: C-reactive protein
EBV: Epstein–Barr virus
ESR: Erythrocyte sedimentation rate
FeSO4: Ferrous sulfate
HIV: Human immunodeficiency virus
HLA: Human leukocyte antigen
HObs: Human osteoblasts
IL-6: Interleukin-6
MRI: Magnetic resonance imaging
NS1: Nonstructural protein 1
NSAIDs: Nonsteroidal anti-inflammatory drugs
OPG: Osteoprotegerin
RANKL: Receptor activator of nuclear factor kappa-B ligand
RRV: Ross River virus
